# Safety and Accuracy of the Freehand Placement of C7 Pedicle Screws in Cervical and Cervicothoracic Constructs

**DOI:** 10.7759/cureus.5304

**Published:** 2019-08-02

**Authors:** William Clifton, Christopher Louie, David B Williams, Aaron Damon, Conrad Dove, Mark Pichelmann

**Affiliations:** 1 Neurosurgery, Mayo Clinic, Jacksonville, USA; 2 Aviation Medicine, Marine Corps Air Station Beaufort, Beaufort, USA; 3 Neurosurgery, Mayo Clinic, Rochester, USA

**Keywords:** cervical pedicle screw, freehand, vertebral artery, spinal instrumentation, cervical fusion

## Abstract

Background: Cervical pedicle screws are advantageous in their biomechanical stability within cervical and cervicothoracic constructs. The seventh cervical vertebra contains relatively large pedicles and has a low incidence of vertebral artery localization within the transverse foramina. The freehand technique of pedicle screw insertion is advantageous in decreasing intraoperative radiation exposure both to the patient and surgeon. In this study, we investigated the safety and accuracy of C7 pedicle screw placement at our institution utilizing an anatomic freehand technique.

Methods and Materials: A retrospective study was performed, and 20 patients were identified who met the inclusion criteria over a five-year period (2013-2018). The C7 pedicle screw placement capability and accuracy were recorded. Accuracy was graded based upon postoperative imaging on a Grade 0-3 scale for breach assessment. Any neurologic complications related to screw placement were also recorded.

Results: Successful pedicle screw placement occurred in 90% of attempts (36/40). The overall screw accuracy rate was 89% (32/36). There were four minor breaches (Grade 1) identified on CT, without neurologic complications. The fusion rate in our cohort for patients with follow up greater than eight months was 100%.

Conclusions: In our patient series, the freehand technique of C7 pedicle screw placement utilizing a small laminotomy with direct pedicle palpation appears to be a safe and accurate method for screw placement, and provides adequate biomechanical stability for cervical and cervicothoracic construct fusion.

## Introduction

Fixation options for the subaxial cervical vertebrae include lateral mass, translaminar, transfacet, and pedicle screws [1]. Pedicle screw placement is biomechanically advantageous due to increased pullout strength of instrumentation [2]. The location of the vertebral artery with respect to the pedicle in the subaxial cervical spine poses a challenge for screw placement [3-4]. At C7, however, the vertebral artery enters the transverse foramen for a small percentage of the time (10%-15%), and thus placement of a pedicle screw at this level poses less of a risk to arterial injury [5]. There are multiple techniques for the placement of C7 pedicle screws, including freehand, CT-guided navigation, and fluoroscopic guidance [6]. The freehand method decreases intraoperative radiation exposure and operative times, however, requires technical acumen and detailed anatomical knowledge of the cervicothoracic junction [7-8]. Familiarity with the freehand method for C7 pedicle instrumentation is an important technique to possess in the surgeon’s armamentarium in the case of navigation equipment failure or incorrect registration. This technique can be guided by direct pedicle palpation through a small laminotomy at the superior aspect of the C7 lamina [9-10]. At our institution we have employed this technique for C7 pedicle screw placement fixation at the cervicothoracic junction. This study aimed to quantify the safety and viability of freehand pedicle screw placement at C7 using this method.

## Materials and methods

With IRB approval, a retrospective review was conducted by two independent reviewers (WC, CL) of all patients undergoing cervical or cervicothoracic fusion by a single surgeon (senior author) at our institution and it was performed over a five-year period (2013-2018). Our inclusion criteria for the study were: adult patients (18 years or older) undergoing posterior cervical or cervicothoracic fusion for any indication that included the C7 level using freehand technique and postoperative X-ray or CT, and any intraoperative attempt to place C7 pedicle screws. Exclusion criteria were patients with previous C7 instrumentation undergoing revision surgery, patients who did not have an intraoperative attempt at C7 pedicle screw placement, and instrumentation using navigation or intraoperative fluoroscopy during screw placement.

Primary outcomes were C7 screw placement capacity, accuracy, and any neurologic complications related to screw placement. Secondary outcome measurement was fusion rates for patients with at least eight months of follow up. Fusion was defined as bony bridging between adjacent vertebral segments throughout the entirety of the construct identified on CT or plain film. Accuracy was graded for patients by analysis of postoperative X-ray or CT. For patients with a postoperative CT, accuracy was graded upon previously published scales [11]: for patients with a postoperative plain film, breach rates were identified based upon a previously published method shown to have high sensitivity and specificity for pedicle screw breaches compared with CT [12]. Demographics recorded included patient age, surgery performed, size and length of C7 screws used, and location of the instrumented C7 level in the fusion construct (bottom or middle). Any identifiable intraoperative breaches requiring screw revision at the time of placement or new postoperative neurologic deficits were also recorded. Postoperative imaging modalities to confirm screw placement were recorded.

**Figure 1:**
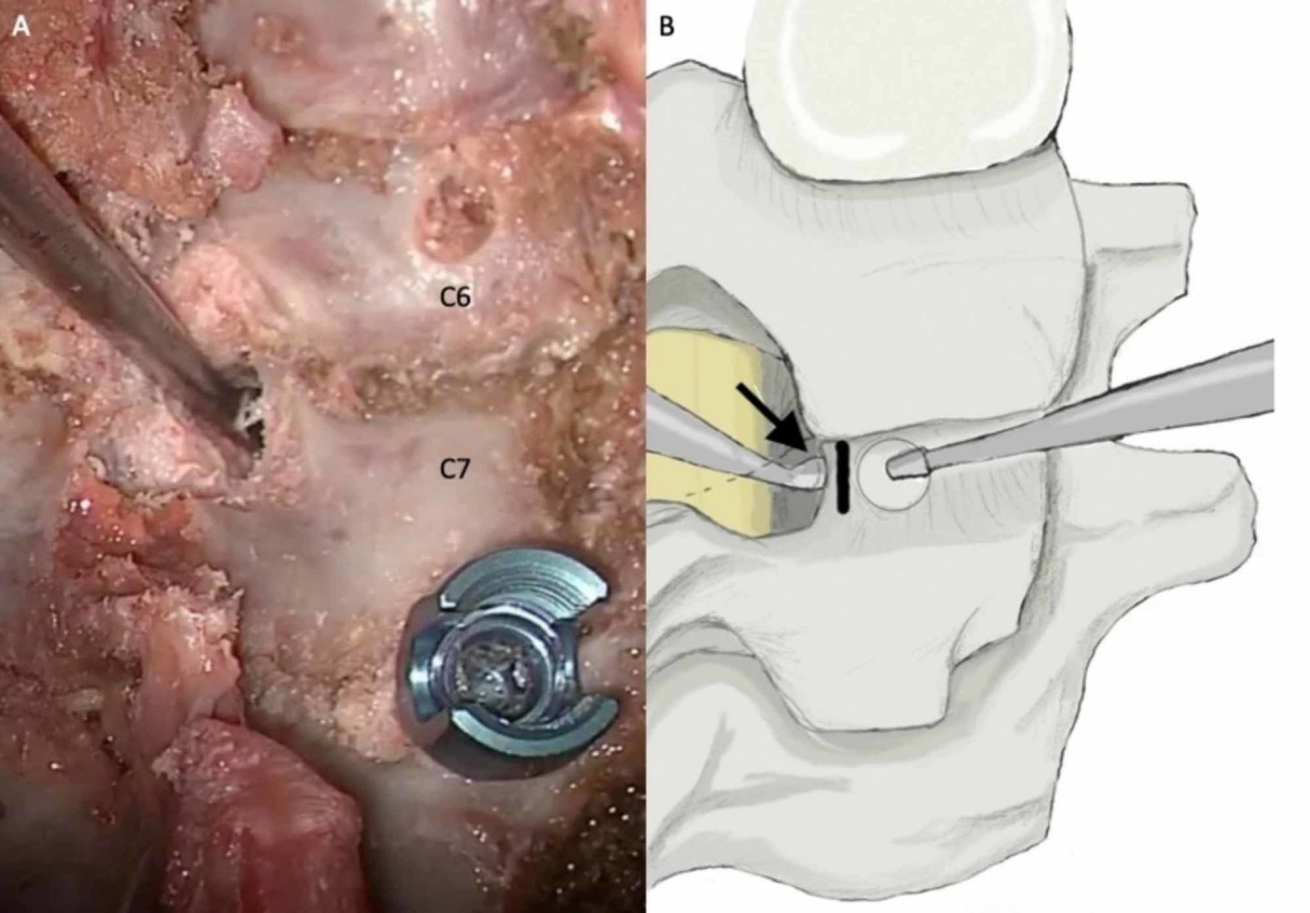
Identification of medial C7 pedicle border. (A) Intraoperative photo of medial C7 pedicle palpation after laminotomy and (B) artist's rendering of the C6-7 level during posterior instrumented cervicothoracic fusion. A curette is placed on the medial border of the C7 pedicle after a small superior laminotomy is performed (black arrow). The black line indicates the projected medial pedicle boundary, which is used for pedicle screw placement trajectory.

**Figure 2:**
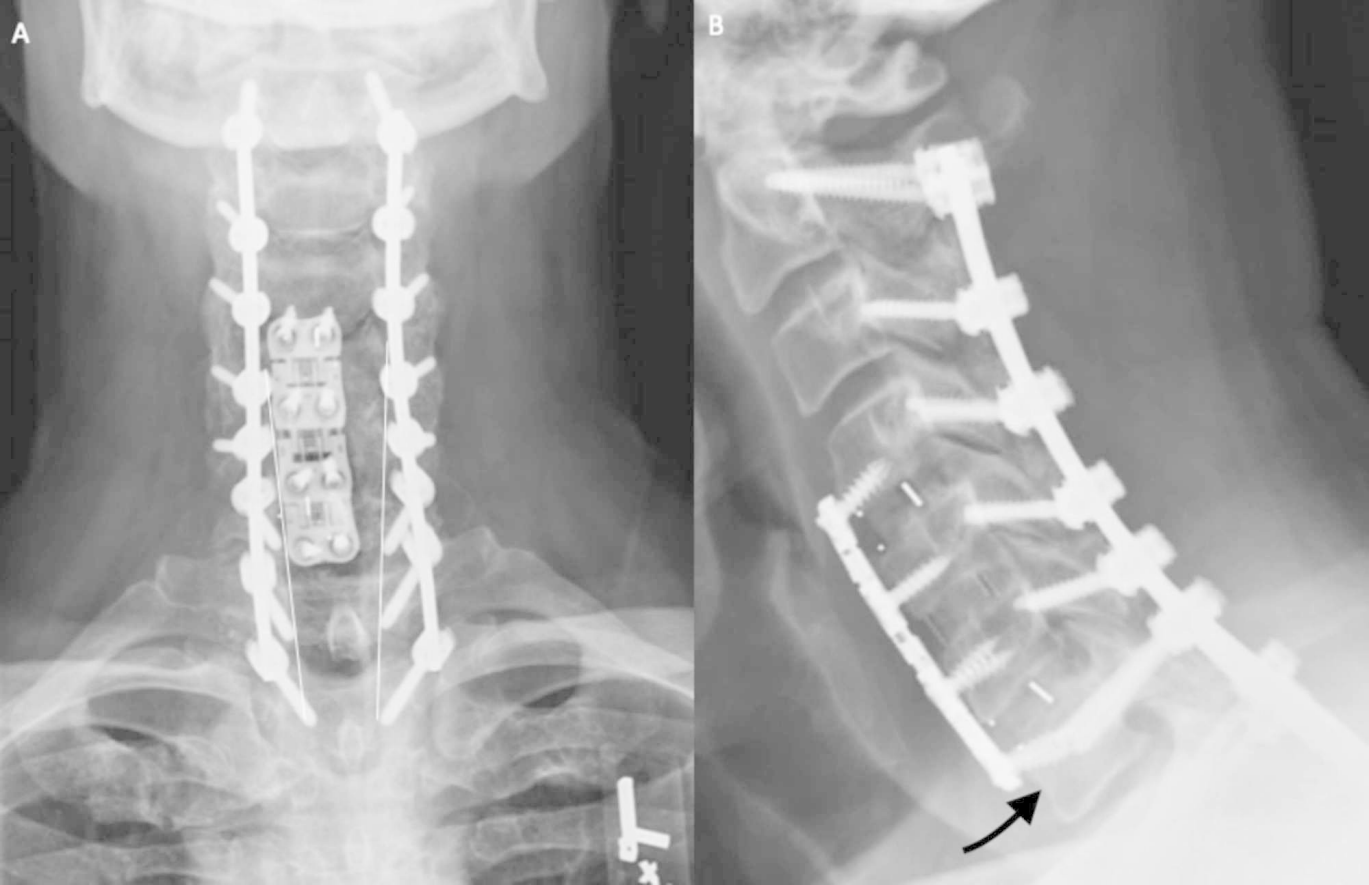
Radiograph breach assessment. (A) The harmonious line conceived by linking the pedicle boundaries at adjacent levels (white lines) allows for assessment of medial and lateral breaches on plain films as previously described by Kim et al. on plain radiographs. (B) Lateral radiographs allow for assessment of anterior breaches by locating the tip of the pedicle screw in relation to the anterior cortex (black arrow).

Details of the utilized operative technique for freehand C7 pedicle screw placement are as follows. A preoperative MRI is obtained on all preoperative patients for surgical planning. The vertebral artery can be easily identified on T2 sequences to determine its course in relation to the C7 pedicle. If a transverse foramen exists at C7 and includes the vertebral artery, then pedicle screws are not placed to avoid neurovascular complications. After complete exposure of the posterior elements of the levels desired to be instrumented, the C7 lateral mass is identified. If the construct is planned to cross the cervicothoracic junction, the thoracic screws are placed first. This aids in starting point planning for the C7 pedicle screw in order to successfully place the rod into the polyaxial heads across the junction. After the thoracic screws are placed, a small laminotomy is performed at the superior border of the C7 lamina (see Figure 1). The pedicle can then be directly palpated. The superior and inferior borders are identified, which provides the cranial-caudal trajectory in order to avoid breaches into the superior or inferior foramen. The medial-lateral trajectory can then be planned based upon the directly palpated medial border of the C7 pedicle, as well as the previously placed thoracic pedicle screw. The starting point is created by a high-speed burr through the posterior cortex. The pedicle is then accessed with a handheld power drill. The drill is initially set to 20 mm, and then can be increased in 2 mm increments until the anterior cortex is found with a ball-tipped probe. The medial, lateral, inferior, superior, and anterior walls are carefully palpated to confirm intraosseous position. A screw measurement is then taken based upon the depth of the ball-tipped probe into the C7 vertebral body. The pedicle tract is then tapped and probed again for any new breaches. The pedicle screw is then placed. The remainder of the subaxial cervical lateral mass screws can then be placed. Antero-posterior and lateral fluoroscopic images are taken intraoperatively to assess hardware position (see Figure 2) [13-14].

## Results

Forty-eight patients were identified that underwent posterior cervical or cervicothoracic fusion by the senior author from 2013 to 2018. Of these patients, 20 met the inclusion criteria. Tables 1-2 contain the pertinent demographics and measured variables for the study. A total of 40 C7 pedicle screw placements in these 20 patients were attempted, with 36 successful (90%). Six patients in our series had constructs ending at C7. There were no observed intraoperative complications or revisions related to screw placement. There were four screw attempts that were not successful. Three were due to screw crowding secondary to structural deformity of the cervicothoracic junction. One screw attempt had a superior breach into the C6-7 foramen, and thus was left out (Patient #17). A lateral mass screw at C7 was placed instead in this instance. There were no neurologic consequences from the initial breach.

**Table 1:** Patient demographics and C7 pedicle screw dimensions. mm, millimeters.

Patient	Age	Procedure	Right pedicle screw size (mm)	Left pedicle screw size (mm)
1	41	C4-C5 decompressive laminectomy; C2-C7 posterior instrumented arthrodesis	4.35 x 25	4.35 x 25
2	53	C3-C7 decompressive laminectomy; C2-T2 instrumented fusion	4.0 x 26	4.0 x 26
3	53	C2-C7 decompressive laminectomy; posterior instrumented cervical arthrodesis	4.35 x 20	4.35 x 25
4	55	Bilateral C4-C5 foraminotomy; Left C7-T1 foraminotomy; Left T1-T2 foraminotomy; C2-T2 instrumented fusion	4.0 x 26	4.0 x 26
5	55	C3-C6 decompressive laminectomy; C2-T1 posterior instrumented fusion	4.0 x 24	4.0 x 26
6	59	C3-4 decompressive laminectomy; C2-T2 instrumented fusion	4.0 x 26	(left out due to crowding)
7	59	C5-C6 decompressive laminectomy; Right C6 foraminotomy; C4-C7 instrumented fusion	4.0 x 24	4.0 x 24
8	60	Right C6-7 revision foraminotomy; C2-T2 instrumented fusion	4.5 x 25	4.5 x 25
9	61	Posterior C7 decompressive laminectomy; C5-T1 posterior instrumented fusion	4.0 x 24	4.0 x 24
10	62	C5-C7 decompressive laminectomy; Right C6 and C7 foraminotomy; Bilateral C8 foraminotomy and resection of synovial cysts; C2-T2 instrumented fusion	4.0 x 26	4.0 x 26
11	64	C3-C6 decompressive laminectomy; C2-T3 instrumented fusion	4.0 x 26	(left out due to crowding)
12	65	C3-C7 decompressive laminectomy; C2-T2 instrumented fusion	4.0 x 26	4.0 x 26
13	66	Bilateral C8 foraminotomy; C7-T1 instrumented fusion	4.0 x 24	4.0 x 24
14	67	Anterior C4, C5, and C6 corpectomy; C3-C7 instrumented fusion; posterior C2-T3 instrumented fusion	(left out due to crowding)	3.5 x 20
15	69	C3-C6 decompressive laminectomy; C2-T2 instrumented fusion	4.0 x 26	4.0 x 26
16	70	Left C7 foraminotomy; C6-C7 laminectomy; C4-C7 instrumented fusion	4.35 x 25	4.35 x 25
17	70	C5, C6, and C7 decompressive laminectomies; C2-T2 posterior instrumented fusion	4.0 x 28	(lateral mass screw placed due to break)
18	71	C2-C7 decompressive laminectomy; C2-C7 instrumented fusion	4.35 x 30	4.35 x 30
19	73	C5 Laminectomy; C3-C7 instrumented fusion	4.0 x 28	4.0 x 28
20	75	C7 decompressive laminectomy; C3-T3 instrumented fusion	4.35 x 25	4.35 x 25

**Table 2:** Patient variables related to C7 pedicle screw placement and assessment. CT, computed tomography; XR, X-ray; L, left; R, right.

Patient	C7 screw intraoperative repositioning required	C7 at bottom of construct	Postoperative CT/XR	Breaches observed	Postoperative neurologic complications	Follow-up to date (months)
1	No	Yes	XR	None	None	25
2	No	No	XR, CT	None	None	13
3	No	Yes	XR, CT	< 2 mm (L-anterior)	None	4
4	No	No	CT, XR	< 2 mm (R-medial)	None	13
5	No	No	XR	None	None	1
6	No	No	XR, CT	None	None	2
7	No	Yes	XR	None	None	12
8	No	No	XR	None	None	6
9	No	No	XR, CT	None	None	13
10	No	No	XR, CT	None	None	11
11	No	No	XR, CT	None	None	12
12	No	No	XR	None	None	25
13	No	No	XR	None	None	12
14	No	No	XR	None	None	2
15	No	No	XR, CT	None	L C5 palsy	2
16	No	Yes	XR, CT	< 2 mm (R-anterior, L-lateral)	None	16
17	No	No	XR, CT	None	None	9
18	No	Yes	XR	None	R C5 palsy	6
19	No	Yes	CT, XR	None	None	3
20	No	No	XR, CT	None	None	8

There were no neurologic complications related to screw placement. Two patients had delayed postoperative C5 palsy not related to instrumentation. All patients received intraoperative X-ray to confirm intraosseous position before procedure end. Eleven patients had a postoperative CT performed within the complete follow-up period. Eight were performed according to the treating surgeon’s standard practice of 8-12 month follow up to assess fusion. Three were performed within the same postoperative hospitalization period. Two of these were performed on the patients with C5 palsies, and one was performed on a patient with beyond expected postoperative incisional pain. None of these three patients had breaches on CT.

The overall accuracy rate for screw placement (Grade 0) was 89% (32/36). There were three patients with four screws with identifiable minor (<2 mm) breaches on CT. Patient #3 had slight bicortical purchase of the left screw. Patient #4 had a small medial breach of the right screw in the proximal portion of the pedicle. Patient #16 had mild anterior bicortical purchase of the right screw, and a small lateral breach on the left screw. None of these patients had neurologic symptoms or complications related to these minor breaches. Fusion rates for patients with at least eight months of follow up was 100% (n = 12). Eight patients received CT evaluation and four received X-ray evaluation of the construct. 

## Discussion

Pedicle screw insertion at C7 is a well-published technique to enhance the biomechanical stability of cervical and cervicothoracic constructs [4, 15-16]. There are numerous methods of pedicle screw insertion. The CT-guided navigation is a commonly used method for patient-specific instrumentation with excellent instrumentation accuracy [6]. The significance of radiation exposure to the patient and practitioner using this approach has been a widely debated topic [8, 17]. Other patient-specific techniques have utilized three-dimensional printing of guide tubes to increase the accuracy of pedicle screw placement at C7 as well as rest of the subaxial cervical spine with good results [18]. This requires labor intensive and time intensive conversion of patient-specific digital imaging files to stereolithography files for printing, making this technique less ideal for emergent cases that do not have substantial time for preoperative planning. Material costs for this method of screw placement are also significant. Anatomical techniques using only posterior element landmarks have been utilized to decrease intraoperative fluoroscopy and radiation exposure [4, 16, 19]. Breach rates using these methods have been reported as high as 13%, however, with a very low rate of neurologic complications [5]. At our institution, we prefer to utilize a small laminotomy as a window to directly palpate the pedicle for ascertainment of the medial, superior, and inferior boundaries. This allows for direct visualization of the pedicle screw insertion trajectory. An advantage of this technique is choosing a pedicle entry point that will allow for pedicle access as well as the ability to properly interact with the superior C6 lateral mass screw head and the inferior T1 pedicle screw head during rod placement across the cervicothoracic junction.

In our study, we did not observe any complications relating to pedicle screw placement. For the 11 patients with postoperative CT, three patients exhibited minor (Grade 1) breaches. Two were bicortically placed anteriorly, one lateral, and one medial (four screws total). For patients with a postoperative plain film, there were no observed breaches based on parameters of radiographic evaluation [12]. Our accuracy rate for screw placement (Grade 0) was 89% (32/36). This rate is similar to other reported studies using freehand technique [3, 4, 9-11, 14].

There were 12 patients in our series that had at least eight months of follow up with imaging to assess fusion. All patients met the criteria for construct fusion. This was not compared with a control, so it is difficult to determine whether there is a significant difference in fusion rates for our cohort with C7 pedicle screws compared with other construct designs. Posterior cervical constructs have a high fusion rate in general as reported in the existing literature [20]. As the primary outcomes for pedicle screw placement in this study were safety and accuracy using a freehand technique, this secondary outcome measure was not investigated further.

There are several limitations to our study. The first is the retrospective method for data acquisition. The second is the heterogenous nature of determining breach rates with CT and plain film. It is likely that very minor breaches (Grade 1) were not able to be identified on plain film evaluation that may have been observed on CT due to resolution differences between the two modalities, possibly creating false negative results. With respect to clinical significance, the Grade 1 breaches observed on CT in our patient cohort did not contact neurovascular structures or produce any patient symptoms. The consequences of minor breaches in pedicle screw placement have been investigated and established in previous publications [5, 14, 19]. As there were no neurologic complications relating to screw placement in our series, the possibility of missed minor breaches did not change patient safety factors or outcomes in this study, indicating clinical insignificance if present.

## Conclusions

Freehand pedicle screw placement at C7 is a safe and viable option for fixation of posterior cervical constructs at the cervicothoracic junction. In the widespread advent of new technologies for instrumentation such as robotic systems and optical navigation, the surgical anatomy of proper C7 pedicle screw placement remains an important and relevant concept to ensure accurate instrumentation independent of technologic availability.
